# Activation of Nucleotide Oligomerization Domain 2 (NOD2) by Human Cytomegalovirus Initiates Innate Immune Responses and Restricts Virus Replication

**DOI:** 10.1371/journal.pone.0092704

**Published:** 2014-03-26

**Authors:** Arun Kapoor, Michael Forman, Ravit Arav-Boger

**Affiliations:** 1 Department of Pediatrics, Johns Hopkins University School of Medicine, Baltimore, Maryland, United States of America; 2 Department of Pathology, Johns Hopkins Medical Institutions, Baltimore, Maryland, United States of America; University of Regensburg, Germany

## Abstract

Nucleotide-binding oligomerization domain 2 (NOD2) is an important innate immune sensor of bacterial pathogens. Its induction results in activation of the classic NF-κB pathway and alternative pathways including type I IFN and autophagy. Although the importance of NOD2 in recognizing RNA viruses has recently been identified, its role in sensing DNA viruses has not been studied. We report that infection with human cytomegalovirus (HCMV) results in significant induction of NOD2 expression, beginning as early as 2 hours post infection and increasing steadily 24 hours post infection and afterwards. Infection with human herpesvirus 1 and 2 does not induce NOD2 expression. While the HCMV-encoded glycoprotein B is not required for NOD2 induction, a replication competent virion is necessary. Lentivirus-based NOD2 knockdown in human foreskin fibroblasts (HFFs) and U373 glioma cells leads to enhanced HCMV replication along with decreased levels of interferon beta (IFN-β) and the pro-inflammatory cytokine, IL8. NOD2 induction in HCMV-infected cells activates downstream NF-κB and interferon pathways supported by reduced nuclear localization of NF-κB and pIRF3 in NOD2 knockdown HFFs. Stable overexpression of NOD2 in HFFs restricts HCMV replication in association with increased levels of IFN-β and IL8. Similarly, transient overexpression of NOD2 in U373 cells or its downstream kinase, RIPK2, results in decreased HCMV replication and enhanced cytokine responses. However, overexpression of a mutant NOD2, 3020insC, associated with severe Crohn's disease, results in enhanced HCMV replication and decreased levels of IFN-β in U373 cells. These results show for the first time that NOD2 plays a significant role in HCMV replication and may provide a model for studies of HCMV recognition by the host cell and HCMV colitis in Crohn's disease.

## Introduction

Infection with human CMV (HCMV), a member of the herpesvirus family, is common in humans. Seroprevalence rates increase with age, reaching 80–90% in individuals older than 80 years [1]. While infection in the normal host is usually asymptomatic, HCMV is a major pathogen in immunocompromised patients and the congenitally-infected newborns [2]–[4]. In these cohorts infection can be severe, persistent, recurrent, or resistant to anti-viral therapy.

Despite being a very common pathogen, around 10–15% of individuals remain HCMV negative for life. HCMV seronegativity may reflect lack of exposure to the virus; alternatively, host genetics may contribute to susceptibility to HCMV infection. Indeed, host genetics can influence susceptibility to human infection and cytokine production by the innate immune system [5]–[8].

Mutations in signaling proteins of the innate immune system have been implicated in the severity of herpesvirus infections [9]. The most studied group of pattern recognition receptors (PRRs) in the setting of HCMV infection has been the Toll-like receptors (TLRs). TLR2 was reported to recognize HCMV and trigger an inflammatory cytokine production [10], [11]. CMV-encoded glycoprotein B (gB) and gH were shown to interact with TLR2 and TLR1 [11]. A single nucleotide polymorphism (SNP) in TLR2 (Arg753Gln) was associated with HCMV disease in a cohort of liver transplant recipients [12].

The role of cytosolic proteins in sensing herpesviruses is gaining significant research interest [13]. The cytoplasmic dsDNA sensor ZBP1 was found important in HCMV-mediated activation of IRF3 and its constitutive overexpression inhibited HCMV replication [14]. Interferon (IFN)-inducible protein, IFI16, inhibited HCMV replication by directly blocking Sp1-mediated transcription of HCMV genes UL54 and UL44, involved in viral DNA synthesis [15]. Amongst the nucleotide-binding oligomerization domain and leucine rich repeat containing receptors (NLRs), NLRC5 was involved in interferon (IFN)-dependent anti-HCMV immune responses. Infection of human fibroblasts with HCMV, but not heat-inactivated virus, induced NLRC5 mRNA within 24 h following infection and knockdown of NLRC5 impaired the upregulation of interferon alpha (IFN-α) in response to HCMV [16]. Involvement of other NLRs in innate immune response to HCMV and the interaction between these receptors in different cell compartments has not been well-studied.

NOD1 and NOD2 are the most widely studied members of the NLR family. These cytoplasmic receptors are highly expressed in monocytes, macrophages, and dendritic cells [17], [18]. NOD1 is also expressed in epithelial cells, and NOD2 expression can be induced in these cells by inflammatory signals [19]. Mutations in NOD2 are strongly associated with Crohn's disease, whereas mutations in NOD1 have been associated with asthma and atopic eczema [20]–[22]. NOD1 recognizes a fragment of peptidoglycan (PGN) containing the dipeptide γ-d-glutamyl-meso-diaminopimelic acid (iE-DAP) produced by Gram-negative and some Gram-positive bacteria. NOD2 recognizes muramyl dipeptide (MDP), present on most types of PGN. Although NOD1 and NOD2 are well-established as intracellular sensors of bacteria [17], [21], [23]–[28], recent studies showed that RNA viruses can also activate NOD2 [29], [30]. NOD2 activation by Respiratory Syncytial Virus (RSV) resulted in its relocalization to the mitochondria and binding to the mitochondrial antiviral-signaling protein (MAVS), a process that was independent of the NOD2 downstream kinase, RIPK2, and resulted in activation of IRF3 and MAVS [29]. The contribution of NOD1 and NOD2 to herpesvirus infections has not been studied.

## Materials and Methods

### Ethics Statement

Clinical isolates of HCMV and herpesvirus 2 (HSV2) were obtained from the microbiology laboratory at Johns Hopkins Hospital with no identifiers that could be linked to a patient. The Johns Hopkins School of Medicine Office of Human Subject Research Institutional Review Board (IRB-X) determined that the research qualified for an exemption.

### Cell culture and Viruses

Human Foreskin Fibroblasts (HFFs) passage 12–16 (ATCC, CRL-2088) and U373 glioma cells (provided by Dr. Gary Hayward, Johns Hopkins Medical Institutions [31]) were grown in Dulbecco's Modified Eagle Medium (DMEM) containing 10% fetal bovine serum (FBS) (Gibco, Carlsbad, CA) in a 5% CO_2_ incubator at 37°C and used for infection with several HCMV strains. One day prior to infection, 8×10^4^ HFFs or U373 cells were seeded on each well of 12-well tissue culture plates. Infection was carried out at multiplicity of infection of 1 PFU/cell (MOI = 1) unless otherwise specified. The pp28-luciferase HCMV Towne strain which expresses luciferase under the control of pp28 late promoter has been described and was shown to correlate well with the classic plaque assay [32]. HCMV strain TB40 with the UL32 gene fused to GFP was obtained from ATCC (VR-1578). Clinical isolates of HCMV and HSV2 were obtained from the microbiology laboratory at Johns Hopkins Hospital with no identifiers that could be linked to a patient. A luciferase-tagged HSV1 (KOS/Dlux/oriS) was provided by Dr. David Leib, Dartmouth Medical School.

### Ultraviolet (UV) inactivation of HCMV

HCMV was UV inactivated by spreading a thin layer of stock suspension in an uncovered six-well tissue culture plate and exposing to a total dose of 720 mJ/cm^2^ in a UV crosslinker (Spectrolinker XL-1000, Spectronics, Westbury, NY) [33]. Luciferase activity was measured in cell lysates of UV inactivated HCMV- infected HFFs 72 hours post infection (hpi), to quantify the level of inactivation. Luciferase units measured from the UV-inactivated HCMV were similar to those measured in the negative control wells, confirming near-complete virus inactivation.

### Chemicals and proteins

MDP was obtained from Sigma Chemicals, (St. Louis, MO) and dissolved in PBS to prepare a stock of 10 mg/ml. Recombinant HCMV gB was purchased from DevaTal Inc. (Hamilton, NJ).

### Plasmids, transfections and virus replication assays

U373 cells were transfected with the following plasmids using Lipofectamine 2000 (Invitrogen, Carlsbad, CA): Human RIPK2 (pcDNA4/HisMax-hRIPK2), human NOD2 (pcDNA4/Hismax-hNOD2) and control pcDNA4/HisMax, (kindly provided by Dr. Michael Davey, Oregon Health and Science University). A pcDNA4-EGFP plasmid was used as additional control. NOD2 3020insC cDNA was PCR amplified from 3020insC-pEF6V5 plasmid (provided by Dr. Jurgen Harder, University Hospital Schleswig-Holstein, Campus Kiel, Germany) and subcloned into BamH1 and Xho1 site of pcDNA4/HisMax plasmid to generate pcDNA4/HisMax-hNOD2 3020insC construct. For amplification of NOD2 3020insC cDNA the following primers were used: 5′ – GGATCCATGGGGGAAGAGGGTGGTTC- 3′ (Forward) and 5′- CTCGAGTCAAAGCAAGAGTCTGGTGTCCC- 3′ (Reverse). Restriction enzyme sites are underlined. The PCR protocol included preheating at 98°C for 30 s, followed by 35 cycles of 98°C (10 s), 60°C (30 s) and 72°C (2 min). PCR products were purified, cleaved by BamH1 and Xho1 and ligated into BamH1 and Xho1 cleaved pcDNA4/HisMax plasmid. The sequence of developed constructs was confirmed by DNA sequencing at the synthesis and sequencing facility, Johns Hopkins University.

Transient transfections of U373 cells were performed in 12-well plates with 1 μg/well of each plasmid using Lipofectamine 2000. After overnight transfection media were changed and cells were allowed to grow for another 24 hours before infection with pp28-luciferase HCMV. Luciferase activity (a measure of late HCMV gene expression) was determined at 96 hpi. We reported that the pp28-luciferase assay correlates well with plaque reduction [32]. DNA replication in HCMV-infected U373 cells and virus DNA yield in supernatants from U373 were measured using real-time PCR as previously below [34]. Virus DNA yield in supernatants from HCMV-infected HFFs were measured using real-time PCR. Virus DNA yield was also measured in supernatants of fresh HFFs after second cycle infection using supernatants from NOD2 knockdown and NOD2 control HFFs.

### Generation of recombinant lentiviral vectors and establishment of stable cell lines

Stable cell lines overexpressing human NOD2 and control plasmids were generated using a doxycycline-inducible TRIPZ lentiviral vector (Open Biosystems, Huntsville, AL). The pMACS Kk hNOD2 HA(C) vector encoding full length human NOD2 (provided by Dr. Atsushi Kitani, NIAID/NIH) was used to prepare NOD2-TRIPZ expression constructs. NOD2 cDNA was subcloned into AgeI and MluI sites of pTRIPZ vector to create pTRIPZ-NOD2 construct. NOD2 cDNA was amplified by PCR using the following primers: 5′-GGGATCCACCGGTCCACCATGGGGGAAGAG-3′ (Forward) and 5′-GGGATCCACGCGTTCATTAAGCGTAGTCTGGGACGTC-3′ (Reverse). Following preheating at 98°C for 30 s, the conditions used for PCR were, 98°C (10 s), 55°C (30 s), 72°C (2 min), for 35 cycles. PCR products were purified, digested with AgeI and MluI, and ligated to pTRIPZ vector to generate hNOD2-TRIPZ construct. The sequence of the new constructs was confirmed by DNA sequencing. hNOD2-TRIPZ and TRIPZ control empty vector were packaged using lentivirus, as described below for the knockdown procedure. To generate stable HFF cell lines expressing hNOD2 or control plasmid, the lentivirus particles were transduced into HFFs. 0.5×10^6^ cells were plated onto T-25 flask, and 40 μl of concentrated virus and Polybrene at final concentration of 8 μg/ml were added to the cells, and incubated for 4 h. 48 h following transduction puromycin (2 μg/ml) containing media was added to culture flasks to select for stably transduced cells. NOD2 over-expressing and control cells were counted and an equal number of cells was seeded into each well prior to infection. An MTT assay (Sigma-Aldrich, St. Louis, MO) was performed to rule out cell toxicity following 48 h doxycyline induction. After the addition of 20 μl/well of MTT (3-(4,5-Dimethyl-2-thiazolyl)-2,5-diphenyl-2H-tetrazolim bromide) (5 mg/ml in PBS), and shaking at 150 rpm for 5 minutes the plates were incubated at 37°C for 3 hours. Conversion of yellow solution to dark blue formazan by mitochondrial dehydrogenases of living cells was quantified by measuring absorbance at 560 nM.

### Lentivirus-mediated Knockdown (KD) of NOD2

Human GIPZ lentiviral shRNAmir constructs (Open Biosystems) were used for NOD2 KD in U373 and HFFs. Four clones (clone id: V2LHS_225438, V3LHS_11832, V3LHS_365839, and V3LHS_365841) targeting different regions of NOD2 mRNA were tested for KD efficiency, and the clone with the best KD efficiency was selected to generate stable cell lines. GIPZ non-targeting control plasmid was used to rule out non-specific effects of shRNAmir constructs. Individual shRNAmir constructs were packaged using lentivirus as described [35]. Briefly, 21 μg of gag/pol, 7 μg of vesicular stomatitis virus glycoprotein, and 7 μg of shRNAmir plasmids were transfected into HEK293 cells using calcium phosphate method. After 48 h the packaged lentivirus particles were concentrated from the medium. The supernatant was filtered and centrifuged at 1750 g for 30 min at 4°C in Amicon Ultra (Ultracel 100 k, Millipore). After centrifugation, 2 ml of cold PBS was added and the tubes were centrifuged again for 20 min at 4°C. The concentrated virus was stored at −80°C until used. Lentivirus particles containing shRNAmir were transduced into HFFs or U373 cells. 0.5×10^6^ cells were plated onto T-25 flask and 40 μl of concentrated virus and polybrene (final concentration, 8 μg/ml) were added to the cells, and incubated for 4 h. Following transduction puromycin (2 μg/ml) was added to select for stably transduced cells. Control HFFs and NOD2 KD HFFs were counted and equal number of cells was plated into each well prior to infection.

### RNA isolation and real time quantitative reverse transcriptase (qRT) PCR

Total RNA was isolated from cultured cells using RNeasy Mini kit (Qiagen, Georgetown, MD) according to manufacturer's instructions. RevertAid first strand cDNA synthesis kit (Fermentas life sciences, Cromwell Park, MD) was used to synthesize first strand cDNA from total RNA using oligo-dT primers. Negative reverse-transcriptase (-RT) reactions were included to ensure the specificity of qRT-PCR reactions. Synthesis of first strand cDNA from mRNA template was carried out at 42°C for 1 h. Quantitative RT-PCR (qRT-PCR) was performed using specific primers and SYBER green (Fermentas life science) with two-step cycling protocol (95°C for 15 s, 60°C for 1 min). Reactions were performed in triplicates and GAPDH was used as internal control. mRNA levels in HCMV-infected cells were normalized to the mRNA produced in non-infected HFFs in addition to the internal normalization of each sample to GAPDH. The primers and gene targets appear in Table 1.

**Table 1:** Primers used for qualitative and quantitative real-time PCR.

Gene	Forward	Reverse
NOD1	5′-CCTAGACAACAACAATCTCAACGACTA-3′	5′-TTTACCCCACCGTCAGTGATC-3′
NOD2	5′-GCCACGGTGAAAGCGAAT-3′	5′-GGAAGCGAGACTGAGCAGACA-3′
IL8	5′-TGCAGCTCTGTGTGAAGGTGCAGT-3′	5′-CAGTGTGGTCCACTCTCAATCACTC-3′
IFN-β	5′-GATTCATCTAGCACTGGCTGG-3′	5′-CTTCAGGTAATGCAGAATCC-3′
ISG15	5′-GCT CCA TGT CGG TGT CAG AG-3′	5′-CTC GAA GGT CAG CCA GAA CAG-3′
Viperin	5′-CAA GAG GAG AAA GCA GCA GC-3′	5′-CAG GAG ATA GCG AGA ATG TCC-3′
GAPDH	5′-TTGGTATCGTGGAAGGACTC-3′	5′- ACAGTCTTCTGGGTGGCAGT-3′
GAPDH (RT)	5′-CAAGGTCATCCATGACAACTTTG-3′	5′-GTCCACCACCCTGTTGCTGTAG-3′

### Real-time PCR

HCMV DNA replication in cells and virus DNA yield in supernatants were quantified using a real-time PCR of the highly conserved US17 as previously described [32], [36].

### SDS-polyacrylamide gel electrophoresis and immunoblot analysis

Cell lysates containing equivalent amount of proteins were mixed with an equal volume of sample buffer (125 mM Tris-HCL, pH 6.8, 4% SDS, 20% glycerol and 5% *β*-mercaptoethanol) and boiled at 100°C for 10 min. Denatured proteins were resolved in Tris-glycine polyacrylamide gels (10–12%) and transferred to polyvinylidine difluoride (PVDF) membranes (Bio-Rad Laboratories, Hercules, CA) by electroblotting. Membranes were incubated in blocking solution [5% non-fat dry milk and 0.1% Tween-20 in PBS (PBST)] for 1 hr, washed with PBST, and incubated with antibody at 4°C overnight. Membranes were washed with PBST and incubated with horseradish peroxidase-conjugated secondary antibodies in PBST for 1 hr at room temperature. Following washing with PBST, protein bands were visualized by chemiluminescence using SuperSignal West Dura and Pico reagents (Pierce Chemical, Rockford, IL). Antibodies for HCMV included: mouse anti- human CMV IE1 & IE2 (MAB810) (Millipore, Billerica, MA, 1∶2,000), mouse anti-human CMV UL83 (pp65) (Vector Laboratories Inc., Burlingame, CA, 1∶2,000), mouse anti-human CMV UL44 (10E8), mouse anti-human β-actin (Sigma, 1∶5,000). For detecting NOD2, mouse anti-human NOD2 (2D9) antibody (Novus Biologicals, Littleton, CO, 1∶2000) and rabbit anti human-NOD2 (H-300) antibody (Santa Cruz Biotechnology Inc, Santa Cruz, CA, 1∶2000) were used. Mouse anti-NF-κB (p65, Sc-8008), rabbit anti-IRF3 antibody (FL-425, Sc-9082) (Santa Cruz biotechnology Santa Cruz, CA, 1∶2,000), and rabbit anti-Histone H3 (D1H2, #4499) antibody (Cell Signaling Technology, 1∶2000) were used for detection of these proteins in cytoplasmic and nuclear extracts.

### Preparation of cytosolic and nuclear extracts

Cytoplasmic and nuclear fractions were isolated from HFF-shNOD2 and HFF-control cells as previously reported with minor modifications [37]. Extracts were prepared from HCMV-infected or mock-infected cells at 24 hpi. Briefly, cells were washed twice with ice-cold phosphate-buffered saline (PBS) and resuspended on ice for 15 min in buffer A containing 10 mM HEPES (pH 7.9), 10 mM KCl, 0.1 mM EDTA, 1 mM dithiothreitol (DTT), protease and phosphatase inhibitors. Cells were then lysed by adding 0.1% NP40 and cytosolic supernatants were obtained by centrifugation at 10,000 rpm for 30 sec. Crude nuclei were washed twice with buffer A to prevent cytosolic contamination, and the nuclear proteins were extracted by resuspending cell pellets with buffer C containing 20 mM HEPES (pH 7.9), 400 mM NaCl, 1 mM EDTA, 1 mM DTT, protease and phosphatase inhibitors. The mixture was incubated for 15 min with vigorous shaking on rocker at 4°C and then centrifuged at 14,000 rpm at 4°C for 10 min to obtain the nuclear proteins. Protein concentration was determined using BCA protein assay reagent kit (Pierce Chemical, Rockford, IL).

### ELISA

Human interferon β (IFN-β) specific ELISA kit (PBL InterferonSource) was used to measure levels of secreted IFN-β from HFF-control and HFF-NOD2 overexpressing cells according to manufacturer's instructions.

### Yield Assay

Human lung fibroblast cells, MRC5, (Diagnostic Hybrids, Athens, OH, 51-0600) were used to perform a virus yield assay. Cells were seeded into 12 well plates (2×10^5^ cells/well) and infected using cell free supernatants collected 3 days post infection (dpi) from HCMV-infected HFF-GIPZ (control) or HFF-shNOD2 cells (second cycle infection). After 90 minute adsorption, media were aspirated, and DMEM containing 0.5% carboxymethyl-cellulose and 4% fetal bovine serum (FBS) were added into duplicate cells. After incubation at 37°C for 8 days the overlay was removed and plaques were counted after crystal violet staining.

### Statistical Analysis

Data were expressed as mean ± SD of three or more independent experiments. The data were analyzed by one-way ANOVA comparisons between different groups with significance value set at *P*<0.05.

## Results

### HCMV infection results in significant induction of NOD2 expression

**Figure 1:**
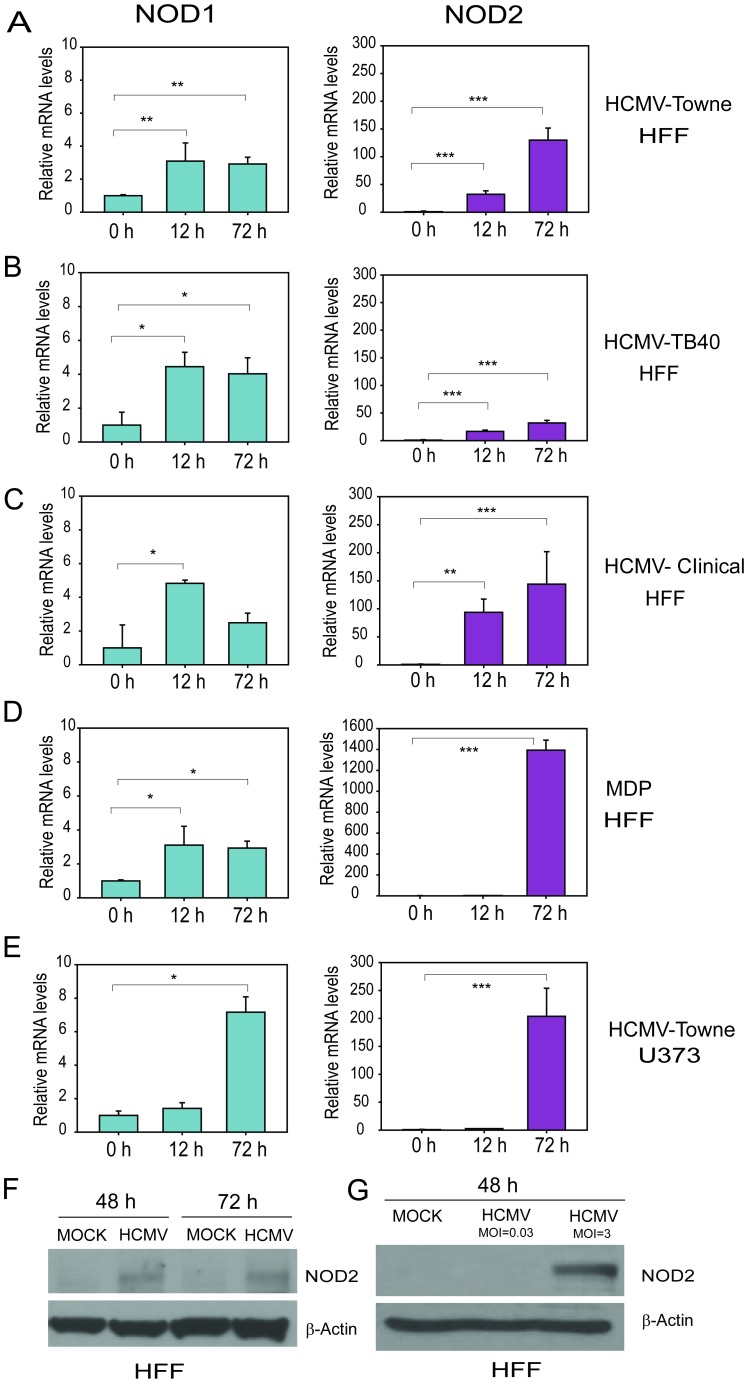
HCMV infection induces NOD2 mRNA and protein in HFFs and U373 cells. **A.** HFFs were infected with HCMV Towne strain and levels of NOD1, NOD2 and Glyceraldehyde 3-phosphate dehydrogenase (GAPDH) mRNAs were measured by qRT-PCR at indicated time points. **B**. HFFs were infected with HCMV-TB40 and levels of NOD1, NOD2 and GAPDH mRNAs were measured by qRT-PCR at indicated time points. **C**. HFFs were infected with a clinical isolate of HCMV and levels of NOD1, NOD2 and GAPDH mRNAs were measured by qRT-PCR at indicated time points. **D**. HFFs were treated with MDP (10 μg/ml) and levels of NOD1 and NOD2 mRNA were measured as in **A, B and C,** **E**. U373 glioma cells were infected with HCMV Towne strain and levels of NOD1, NOD2 and GAPDH mRNAs were measured by qRT-PCR at indicated time points. **F.** HFFs were infected with HCMV (Towne) at MOI of 1 PFU/cell and levels of NOD2 protein and β-actin were determined 48 and 72 hpi. **G.** HFFs were infected with HCMV (Towne) strain at MOI of 0.03 or 3 PFU/cell and levels of NOD2 protein and β-actin were determined at 48 hpi. Quantitative data represent mean values (±SD) of triplicate determinations from three independent experiments (*p<0.05, **p<0.01, ***p<0.001, one-way ANOVA test).

mRNA levels of NOD1 and NOD2 were measured by qRT-PCR in HCMV-infected HFFs. The following HCMV strains were used for infection of HFFs at MOI of 1 PFU/cell: the laboratory-adapted strain, Towne, the endotheliotropic TB40-GFP strain (ATCC VR-1578), and a clinical isolate of HCMV. Infection with all three viruses (Towne, TB40 and a clinical isolate) resulted in robust induction of NOD2 transcripts at 12 and 72 hours post infection (hpi), while in non-infected HFFs NOD2 mRNA was undetectable (Fig. 1A, B and C). Treatment of non-infected HFFs with MDP, a positive control for NOD2 induction [38], resulted in two fold induction of NOD2 expression at 12 h, and a potent NOD2 induction (1400-fold) at 72 h (Fig. 1D). Similarly, infection of U373 glioma cells with Towne HCMV resulted in significant upregulation of NOD2 at 72 hpi (Fig. 1E). In contrast to NOD2, NOD1 was already expressed in non-infected HFFs, and there was a modest increase (∼3–7 fold) in NOD1 transcripts at 12 and 72 hpi (Fig. 1A–E), depending on the HCMV strain and cell type used. NOD2 protein was upregulated in HCMV-infected HFFs at 48 and 72 hpi (Fig. 1F) and its expression was MOI dependent (Fig. 1G).

### Infection with Herpesvirus (HSV1 and HSV2) does not induce NOD2

To determine whether NOD2 could be induced by other herpesviruses, HFFs were infected with herpesvirus 1KOS/Dlux/oriS and a clinical isolate of HSV-2 at MOI of 1 and 0.1, respectively. NOD1 and NOD2 transcripts were quantified at 2, 4, 6, 8 and 24 hpi in HSV-1 infected HFFs using qRT-PCR. There was no significant change in NOD1 expression between non-infected and HSV1-infected HFFs (Fig. S1A). NOD2 was again undetectable in non-infected HFFs, but was not induced after infection with HSV1. Similarly, there was no significant increase in NOD1 expression in HSV2-infected HFFs at 4 and 24 hpi as compared to non-infected HFFs (Fig. S1B) and NOD2 was undetectable in both non-infected and HSV2-infected HFFs.

### NOD2 is induced early after HCMV infection

**Figure 2:**
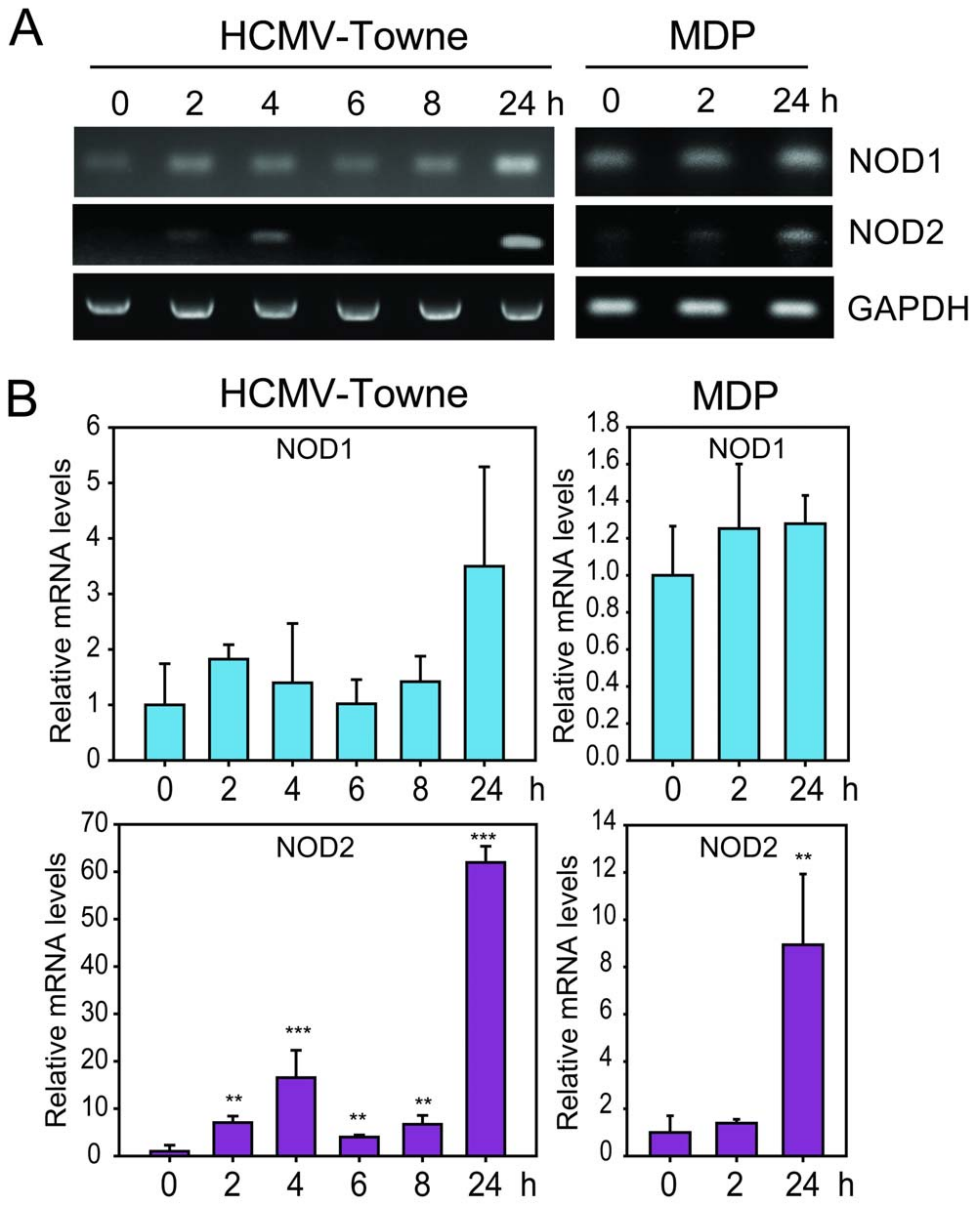
Kinetics of NOD1 and NOD2 transcripts in HCMV-infected cells. **A. B**. HFFs were infected with HCMV Towne strain or treated with MDP (10 μg/ml) and levels of NOD1, NOD2 and GAPDH mRNAs were measured by RT-PCR (A) and qRT-PCR (B) at indicated time points. The data shown are the average of three experiments ± SD (*p<0.05, **p<0.01, ***p<0.001, one-way ANOVA test).

To understand the kinetics of NOD2 induction early after HCMV infection a time-course experiment was performed in HFFs. NOD1 and NOD2 transcripts were quantified starting from the time of HCMV infection and then at 2, 4, 6, 8, and 24 hpi (Fig.2 A, B). MDP was used as positive control for NOD2 induction. Upregulation of NOD2 mRNA was observed as early as 2 hpi and increased significantly at 24 hpi. NOD2 transcripts remained elevated at 72 hpi (Fig. 1 A–C). A modest increase in NOD1 transcripts (up to 5-fold) was observed at 24 hpi.

### Glycoprotein B alone cannot induce NOD2 expression

**Figure 3:**
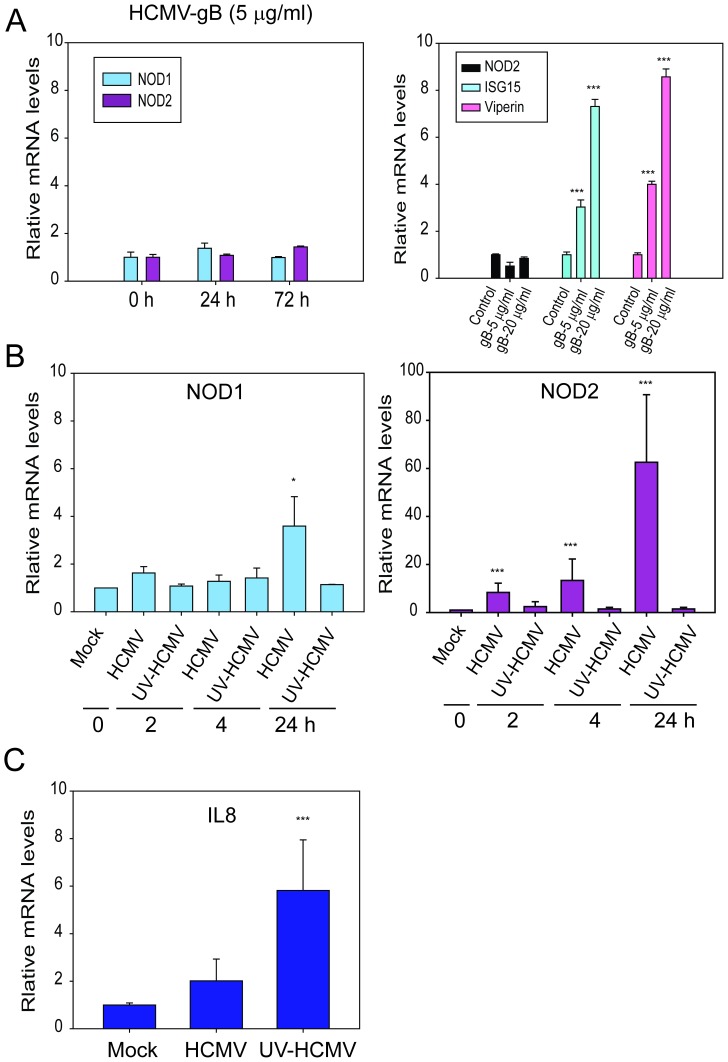
HCMV-encoded glycoprotein B (HCMV-gB) and UV-inactivated virus cannot induce NOD2. **A**. Left- HFFs were treated with recombinant HCMV-gB (5 μg/ml) for 24 h and 72 h and levels of NOD1 and NOD2 mRNA were measured by qRT-PCR. Right- HFFs were treated with recombinant HCMV-gB (5 μg/ml or 20 μg/ml) for 24 h and levels of NOD2, ISG15 and viperin mRNA were measured by qRT-PCR. **B**. HFFs were infected with HCMV Towne or a UV-inactivated HCMV Towne for 2, 4, and 24 h and levels of NOD1 and NOD2 mRNA were measured by qRT-PCR. IL8 levels were measured as control at 4 hpi. Quantitative data represent mean values (±SD) of triplicate determinations from three independent experiments (*p<0.05, **p<0.01, ***p<0.001, one-way ANOVA test).

Since HCMV-encoded gB was reported to bind to and activate TLR2 we tested whether it was required for NOD2 induction [10]. HFFs were treated with purified HCMV gB protein (5 μg/ml or 20 μg/ml, DevaTal, Hamilton, NJ) and levels of NOD1 and NOD2 were measured at 24 and 72 hpi using qRT-PCR. Levels of NOD1 were unchanged in untreated vs gB-treated HFFs. Treatment with gB alone did not induce NOD2 transcripts (Fig. 3A), but interferon inducible gene 15 (ISG15) and viperin were induced by gB.

### UV-inactivated virus is unable to induce NOD2

To determine whether an intact HCMV genome was essential for NOD2 induction a UV-inactivated virus which could bind to the cells but could not replicate was used. The UV-inactivated virus did not induce NOD2 or NOD1 expression, suggesting an intact HCMV genome was required for induction of NOD2 (Fig. 3B). IL8 was induced (6–8 fold) using the UV-inactivated HCMV at 4 hpi, more efficiently than its induction by HCMV, in agreement with previous reports that suggested the induction of IL8 mRNA could be prevented by newly synthesized viral gene products [39].

### Overexpression of NOD2 results in decreased HCMV replication and enhanced antiviral and pro-inflammatory cytokine responses

**Figure 4:**
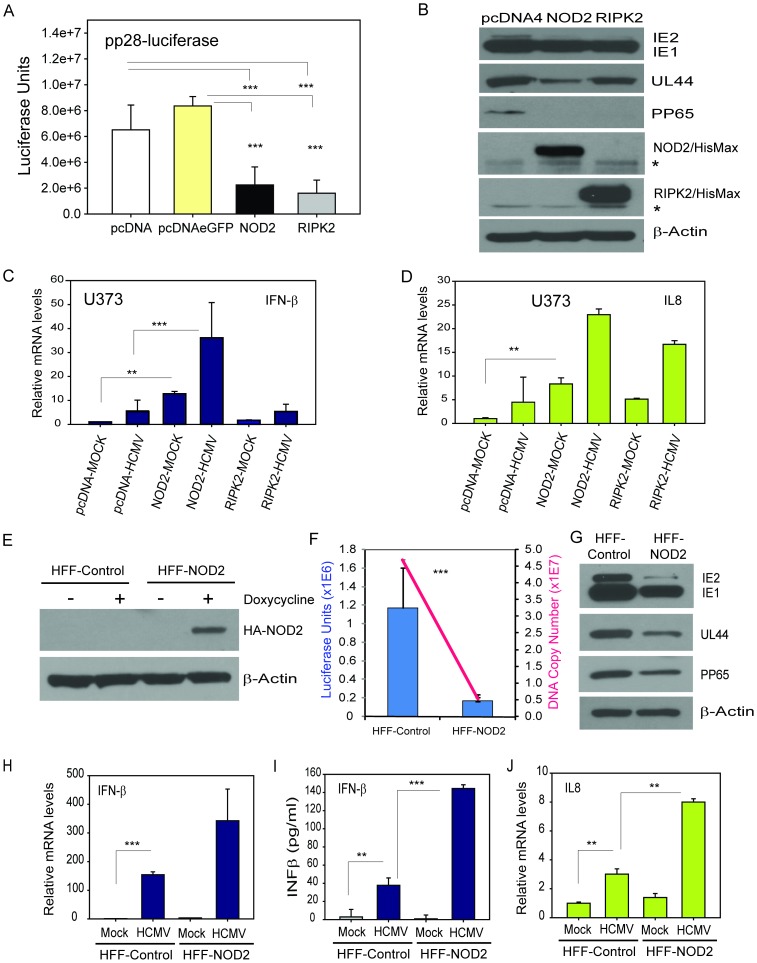
Overexpression of NOD2 restricts HCMV replication and induces antiviral and pro-inflammatory cytokines. **A**. U373 cells were transiently transfected with pcDNA4/HisMax, pcDNA4/EGFP, pcDNA4/HisMax-hNOD2 or pcDNA4/HisMax-hRIPK2 plasmid. 24 h after transfection cells were infected with pp28-luciferase HCMV. Luciferase activity was measured in cell lysates at 72 hpi. **B**. Cell lysates from 4**A** were used to determine protein expression of HCMV-immediate early (IE1/IE2), early (UL44), and late (pp65) genes. Levels of NOD2 and RIPK2 proteins were measured to confirm NOD2 overexpression; β-actin served as loading control. Western blot data are representative of three independent experiments. Asterisks (*) denote endogenous NOD2 and RIPK2 proteins. **C**–**D.** U373 cells were transiently transfected with pcDNA4/HisMax, hNOD2 or hRIPK2 plasmid. 24 h after transfection cells were infected with HCMV Towne and total RNA was isolated at 72 hpi. Levels of IFN-β and IL8 mRNAs were measured by qRT-PCR in non-infected (mock) and HCMV-infected (HCMV) cells. **E.** HFFs stably expressing empty vector (HFF-control) or HA-tagged NOD2 (HFF-NOD2) were either untreated or treated with 2 μg/ml doxycycline and expression of NOD2 was determined at 48 h using anti-HA antibody. **F.** HFFs stably expressing empty vector (control) or NOD2 (HFF-NOD2) were induced with doxycycline for 24 h followed by infection with HCMV Towne. Cells were incubated in doxycycline containing media and cell-free supernatants were collected at 96 hpi. Virus progeny released into the supernatants were quantified after infection of fresh HFFs (second cycle) with equal amount of supernatants from control or NOD2-overexpressing HFFs using luciferase assay at 3 dpi (Y-axis on left, in blue) and by real-time PCR (Y-axis on right, in red) in the supernatants of newly-infected HFFs (Fig. 4F). Viral protein expression was determined in newly-infected HFFs at 3 dpi (Fig. 4 G). **H, J.** Levels of IFN-β and IL8 mRNAs were determined in HFFs-control or HFF-NOD2 cells using qRT-PCR at 96 hpi. **I.** Levels of IFN-β secreted into the media from HFF-control and HFF-NOD2 cells (first cycle of infection) were measured at 24 hpi using IFN-β ELISA kit. The data shown are the average of three experiments ± SD (*p<0.05, **p<0.01, ***p<0.001, one-way ANOVA test).

HCMV induces multiple cellular genes to achieve efficient replication. We hypothesized that if NOD2 had a role in HCMV recognition and was not simply an induced gene amongst many other cellular genes, then its overexpression or KD would result is restricted or enhanced virus replication, respectively, along with changes in antiviral and pro-inflammatory cytokine responses. U373 glioma cells were initially used for transfections because the efficiency of transient transfection followed by HCMV infection in these cells was found to be high. Cells were transfected with human pcDNA4/HisMax-NOD2 or the pcDNA4/HisMax-RIPK2 (a critical kinase downstream of NOD2) plasmids and control plasmids pcDNA4/HisMax and pcDNA4-EGFP. 24 h following transfection cells were infected with HCMV at MOI = 1 and luciferase activity was measured at 72 hpi. NOD2 overexpression resulted in ∼70% reduction of pp28 gene expression (Fig. 4A), consistent with significant inhibition of HCMV replication. In NOD2-transfected HCMV-infected cells the expression of HCMV immediate early 2 (IE2) and the early protein UL44 were significantly reduced as compared to control pcDNA4-transfected HCMV-infected cells. The expression of the late HCMV protein pp65 was completely undetectable in NOD2 overexpressing cells (Fig. 4B). Overexpression of RIPK2 resulted in significant inhibition of pp28-luciferase activity and pp65 expression, suggesting the effect of NOD2 in HCMV-infected cells may involve at least in part RIPK2 activity. Overexpression of NOD2 and RIPK2 was confirmed by western blot using anti-NOD2 and anti-RIPK2 antibodies, respectively (Fig. 4B). Levels of IFN-β and the inflammatory cytokine IL8 were measured by qRT-PCR in NOD2-, RIPK2- and control pcDNA4 plasmid-transfected cells. There was approximately ten- and three-fold increase in expression of IFN-β in NOD2 and RIPK2-overexpressing-HCMV infected cells as compared to HCMV infected cells transfected with pcDNA4 plasmid (Fig. 4C) at 72 hpi. Levels of IL8 mRNA were upregulated by approximately five- and three-fold in NOD2 and RIPK2-overexpressing-HCMV infected cells as compared to HCMV-infected cells transfected with pcDNA4 control plasmid (Fig. 4D) at 72 hpi. These results suggest that the effects of NOD2 on HCMV may involve both IFN-β and IL8.

To further understand the role of NOD2 in regulating HCMV replication, an inducible lentiviral pTRIPZ-based vector overexpressing HA-tagged NOD2 was generated in HFFs (HFF-NOD2). HFFs overexpressing control pTRIPZ empty vector (HFF-control) were also generated. NOD2 expression was confirmed by western blot analysis, performed after 48 h induction with doxycycline, and using anti-HA antibody (Fig 4E). β-Actin was used as a loading control. HCMV replication was next measured in HFF-NOD2 and HFF-control cells. Doxycycline (2 μg/ml) induction was performed 24 h before infection with HCMV pp28-luciferase at an MOI of 0.5. Cells were counted and equal number of cells from each condition was seeded into wells prior to infection. To rule out potential toxicity secondary to induction, a MTT assay was performed 48 h after doxycycline induction and revealed no effect on cell viability. Cell-free supernatants were collected from infected HFFs-NOD2 and infected HFFs-control cells 4 days post infection (dpi) and were used for a second cycle infection of fresh HFFs at equivalent volumes. HCMV replication was then measured in the newly-infected HFFs using pp28-luciferase, virus DNA yield in supernatants of newly-infected HFFs and by western blots for HCMV proteins IE1, UL44 and pp65 (Fig. 4F, G). At least 80% decrease was observed in luciferase expression and virus DNA yield in the second cycle of newly-infected HFFs using supernatants from HFF-NOD2 as compared to HCMV replication in newly-infected HFFs using supernatants from HFF-control cells, suggesting NOD2 induced a cellular immune state that was refractory to HCMV replication. Similarly, there was a significant decrease in the expression of IE1, UL44 and pp65 in HFFs infected with supernatants from HFF-NOD2 transduced cells as compared to HFF-control cells. The observed changes in HCMV replication were associated with changes in the transcripts of IFN-β and IL8, measured at 96 hpi in HFF-NOD2 and HFF-control cells (first cycle). There was approximately two and three-fold increase in the expression of IFN-β and IL8 mRNA, respectively, in HFFs-NOD2 as compared to HFFs-control (Fig. 4H, J). Levels of IFN-β protein secreted into the media from non-infected and HCMV-infected-HFFs-control and HFFs-NOD2 were also measured at 24 hpi using human IFN-β specific ELISA kit. There was approximately five-fold increase in the levels of secreted IFN-β in HCMV infected-HFFs-NOD2 as compared to HCMV infected-HFFs-control (Fig. 4I). In non-infected HFFs-NOD2 the induction of IFN-β and IL8 was similar to that observed in control HFFs (Fig. 4H-J).

### NOD2 knockdown (KD) results in enhanced HCMV replication and decreased levels of cytokines

**Figure 5:**
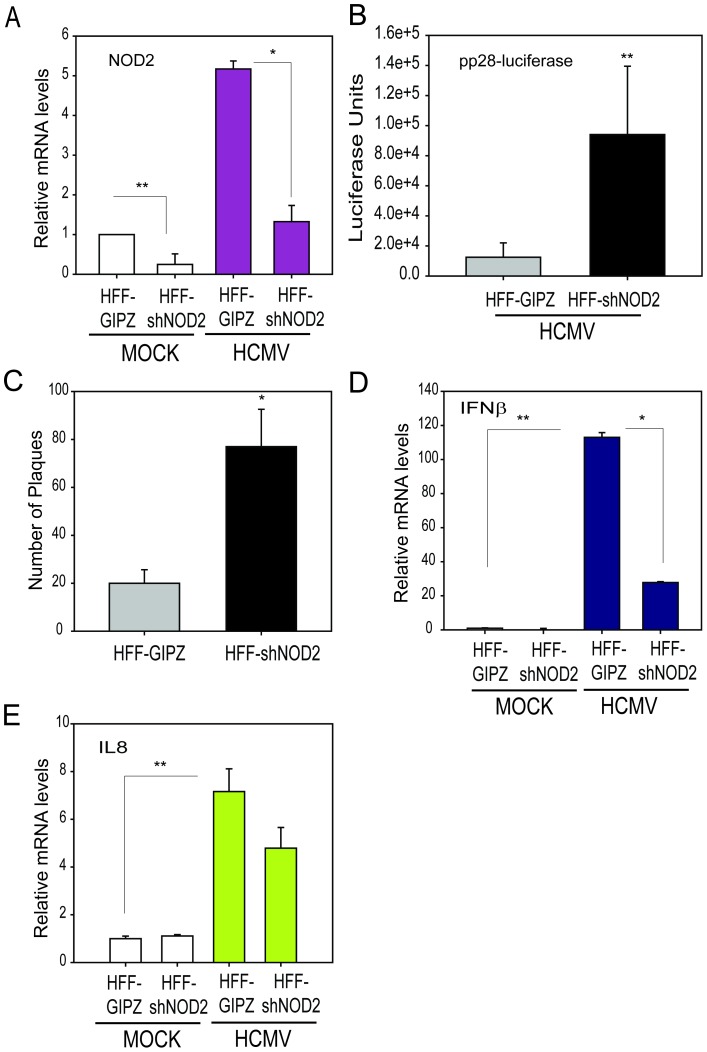
Knockdown of NOD2 results in enhanced HCMV replication in HFFs. **A.** HFF stably expressing control lentiviral vector (HFF-GIPZ) or a lentiviral vector expressing short-hairpin RNA (shRNA) against NOD2 (HFF-shNOD2) were infected with HCMV at MOI = 1, and levels of NOD2 mRNA were measured using qRT-PCR at 72 hpi. **B**. Cell-free supernatants were collected at 96 hpi from HCMV-infected HFF-GIPZ or HFF-shNOD2 cells and used to infect fresh HFFs (second cycle). Luciferase activity was measured at 72 hpi. **C.** Cell-free supernatants were collected at 3 dpi from HCMV-infected HFF-GIPZ (control) and HFF-shNOD2 cells and used to perform a yield reduction assay in fresh HFFs. **D, E.** Levels of IFN-β and IL8 mRNA were measured in cell lysates collected at 72 h from non-infected and HCMV-infected HFF-GIPZ and HFF-shNOD2 cells using qRT-PCR. The data shown are the average of three experiments ± SD (*p<0.05, **p<0.01, ***p<0.001, one-way ANOVA test).

**Figure 6:**
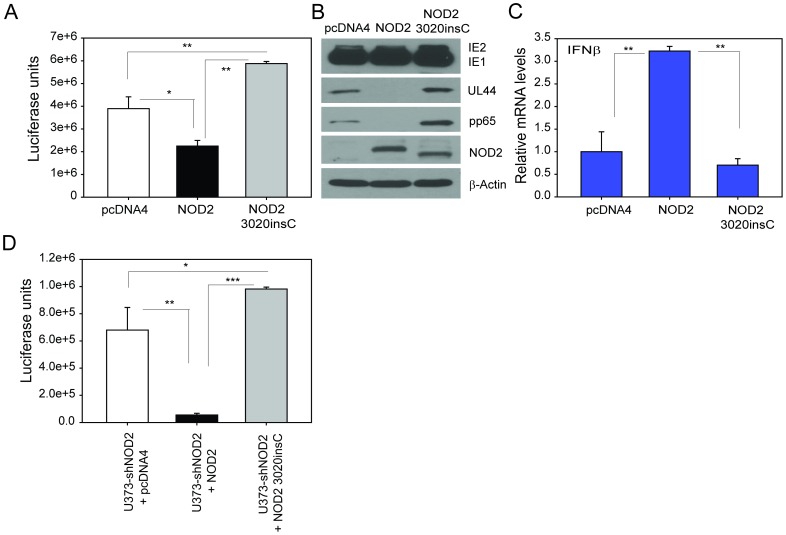
Rescue of NOD2 expression in KD cells restores the restriction of HCMV replication. **A**, **B, C.** U373 cells were transfected with pcDNA4/HisMax, pcDNA4/HisMax-hNOD2 or pcDNA4/HisMax-hNOD2 3020insC and infected with HCMV after 24 h. Virus replication (by luciferase activity), NOD2 expression (by western blot) and expression of cytokines (by qRT-PCR) were measured. **D.** U373-GIPZ or U373-shNOD2 cells were transfected with control plasmid pcDNA4/HisMax (pcDNA4), pcDNA4/HisMax-NOD2 (pcDNA4-NOD2), or pcDNA/HisMax-NOD2 3020insC (NOD2 3020insC) followed by HCMV infection 24 h later. HCMV replication was determined using luciferase assay. Quantitative data represent mean values (±SD) of triplicate determinations from two independent experiments (*p<0.05, **p<0.01, ***p<0.001, one-way ANOVA test).

A short hairpin (shRNA) pGIPZ lentivirus system was used to KD NOD2 expression in the two cell lines: HFFs and U373 cells. NOD2 mRNA levels were decreased by approximately 75% at 72 h in non-infected and HCMV-infected (MOI = 1) NOD2-KD HFFs (HFF-shNOD2) compared to control shRNA (HFF-GIPZ) transduced cells (Fig. 5A). Supernatants from infected HFF-shNOD2 and HFF-GIPZ cells were collected at 96 hpi and used to infect fresh HFFs (second cycle). Luciferase activity, measured in the newly-infected cells at 72 hpi (a measure of infectious progeny released after single cycle infection), was increased by approximately 10-fold in HFF-shNOD2 cells compared to infected HFF-GIPZ control cells (Fig. 5B). Cell-free supernatants collected from HCMV infected-HFF-GIPZ and HFF-shNOD2 cells at 3 dpi were used for virus yield assay. There was approximately 4-fold increase in the number of plaques in cells infected with supernatants from HFF-shNOD2 cells as compared to the cells infected with supernatants from HFF-control cells (Fig. 5C). IFN-β mRNA levels measured in cell lysates were significantly reduced in HCMV infected HFF-shNOD2 cells as compared to infected HFF-GIPZ cells after 72 h (Fig 5D). There was a modest decrease in IL8 transcripts in HCMV infected HFF-shNOD2 cells as compared to infected HFF-GIPZ cells after 72 h (Fig 6E). NOD2-KD in U373 cells showed a similar pattern of enhanced virus replication and decreased cytokine responses to that observed in HFFs, suggesting this is an important mechanism for controlling HCMV replication.

In NOD2-KD U373 cells (U373-shNOD2) NOD2 mRNA levels were decreased by approximately 80% at 72 h in both non-infected and HCMV-infected (MOI = 1) cells (Fig. S2A) compared to control shRNA (U373-GIPZ) expressing cells. HCMV pp28-luciferase expression (measured at 96 hpi), HCMV DNA replication (measured at 48 hpi), and virus DNA yield in supernatants (measured at 96 hpi) from U373-shNOD2 cells and control U373-GIPZ revealed a significant increase in HCMV replication in the U373-shNOD2 cells (Fig. S2B): there was a 9-fold increase in virus DNA yield, 6-fold increase in DNA replication and 5-fold increase in luciferase activity. Quantification of mRNA expression of IFN-β and IL8 revealed a 3.5-fold decrease of IFN-β and 2-fold decrease in IL8 in U373-shNOD2 cells as compared to U373-GIPZ cells (Fig. S2C, D).

### Overexpression of NOD2 mutant (3020insC) results in increased HCMV replication

**Figure 7:**
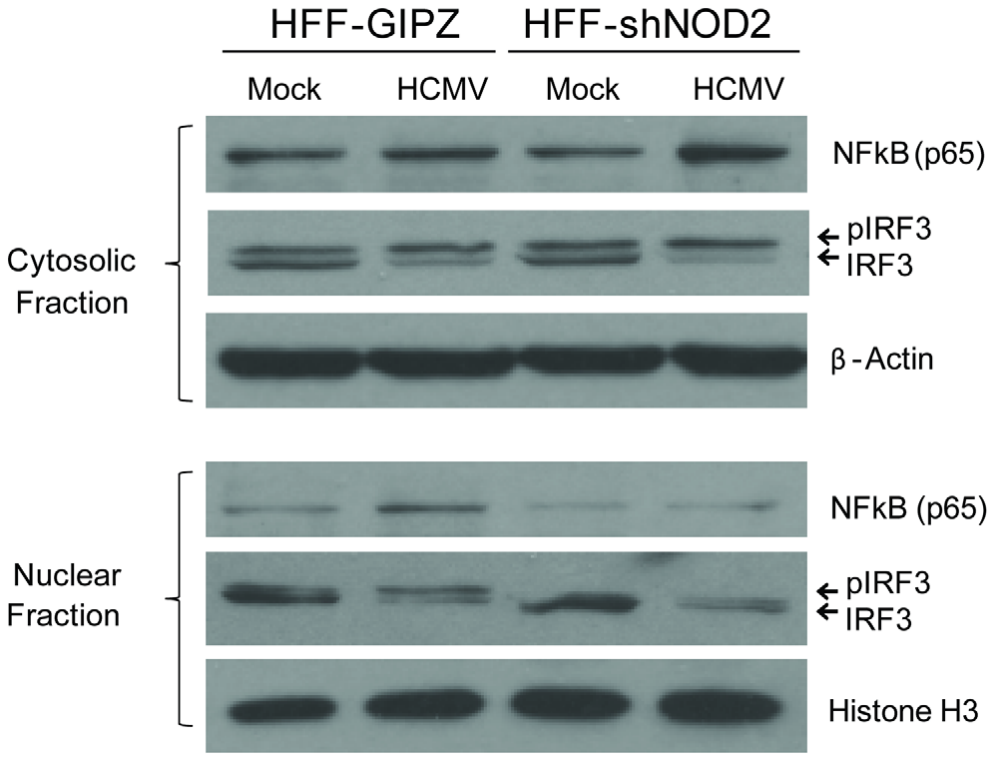
NOD2 KD results in decreased activation of the NF-_K_B and IFN pathways. NOD2 KD HFFs (HFF-shNOD2) and control HFFs (HFF-GIPZ) were infected with HCMV Towne and expression of NF-_K_B (p65) and IRF3 was measured in cytoplasmic and nuclear extracts at 24 hpi. Representative data from three independent experiments are shown.

The frameshift substitution at amino acid 1007 in the NOD2 gene stems from an insertion mutation resulting in a truncated NOD2 and impairing its ability to recognize microbial components. Patients with Crohn's disease that are homozygous for 3020insC demonstrate a much more severe disease phenotype [40]. Since NOD2 KD and its overexpression in HFFs and U373 showed similar effects on HCMV replication and antiviral responses we tested the effect of NOD2 3020insC mutant on HCMV replication in U373 cells. While overexpression of wild-type NOD2 resulted in significantly reduced HCMV replication, overexpression of the NOD2 mutant 3020insC in U373 resulted in increased virus replication (Fig.7A). The expression of NOD2 3020insC was confirmed by western blot (Fig. 7B). IFN-β transcripts were measured in infected U373 cells transfected with pcDNA4, NOD2 or NOD2 3020insC mutant, demonstrating the IFN-β levels were not increased in cells transfected with the NOD2 3020insC, while induced upon transfection with the wild-type NOD2 (Fig. 7C).

### NOD2 rescue in KD-cells restores the ability to restrict HCMV replication

U373 cells stably expressing control GIPZ shRNA (U373-GIPZ) or NOD2 shRNA (U373-shNOD2) were transiently transfected with either control plasmid (pcDNA4/HisMax) or a plasmid expressing NOD2-cDNA (pcDNA4/HisMax-NOD2). Twenty four hours following transfection, cells were infected with HCMV and luciferase activity was measured at 96 hpi. As shown in Fig. S2B, enhanced HCMV replication was observed in U373-shNOD2 cells as compared to virus replication in control U373-GIPZ cells, while overexpression of NOD2 resulted in significant decrease in HCMV replication. Transfection of the NOD2 gene into NOD2-KD U373 cells restored NOD2 function and resulted in restriction of HCMV replication, clearly demonstrating the specific role of NOD2 in HCMV replication (Fig. 7B). However, rescue of NOD2 3020insC in U373-shNOD2 did not lead to restricted HCMV replication (Fig. 7D).

### NOD2 activates the NF-κB and the IFN pathway in HCMV-infected HFFs

Levels of NF-κB and phosphorylated forms of IRF3 were measured by western blot in cytoplasmic and nuclear extracts of HFF-shNOD2 cells and control HFF-GIPZ cells at 24 hpi. Compared to the control HFF-GIPZ cells, in which HCMV infection resulted in NF-κB localization to the nucleus, in the HFF-shNOD2 cells NF-κB remained in the cytoplasm (Fig. 7). Similarly, IRF3 was not activated in HFF-shNOD2 cells. While a phosphorylated form of IRF3 was observed in the nuclei extracted from HCMV-infected HFF-GIPZ cells, the level of nuclear IRF3 in HCMV-infected shNOD2 and its phosphorylated form were significantly decreased. These results suggest NOD2 serves as a central hub, activating both the NF-κB pathway and the IFN pathway following its induction in HCMV-infected cells.

## Discussion

We report for the first time that NOD2 is induced by HCMV and plays a significant role in restricting its replication. Infection of HFFs and U373 glioma cells with laboratory-adapted strains and a clinical isolate of HCMV resulted in a significant induction of NOD2 as early as 2 h after virus infection, required an intact viral genome and persisted throughout a full replication cycle. HSV1 and HSV2 did not induce NOD2 expression in infected HFFs. Overexpression of NOD2 resulted in decreased HCMV replication and enhanced antiviral and pro-inflammatory cytokine responses. NOD2 silencing (or transfection with the NOD2 mutant 3020insC) resulted in enhanced HCMV replication and decreased IFN-β levels. Reintroducing NOD2 into KD cells resulted in restriction of virus replication. Taken together, NOD2 plays a role in recognizing HCMV and restricting its replication. Although prior large scale transcriptomics studies did not report on NOD2 induction in HCMV-infected HFFs, RIPK2, a critical kinase downstream of NOD2 was significantly induced at 24 hpi [39].

Induction of NLRs result in activation of several signaling pathways: 1) The classic pathway is the NF-_K_B. Upon activation, NOD1/2 recruit RIPK2 [17] which promotes the K63-linked polyubiquitylation of the regulator NEMO/IKKγ and activation of the kinase transforming growth factor-β-activated kinase 1 (TAK1), which are prerequisites for the activation of the IKK complex. IKK activation results in degradation of the NF-_K_B inhibitor I_K_Bα and the translocation of NF-_K_B to the nucleus, where transcription of NF-_K_B-dependent target genes occurs. RIPK2 is critical for NOD1- and NOD2-mediated NF-_K_B activation because NOD1 and NOD2 signaling is abolished in RIPK2-deficient cells [41]. In addition to the activation of the NF-_K_B pathway, NOD2 stimulation results in activation of the MAPKs p38, ERK and JNK [42]. 2) Alternative pathways which may or may not require RIPK2 include the induction of type I IFN and autophagy [24], [43]–[45]. Inhibition of HCMV replication appears to involve its downstream kinase, RIPK2, because overexpression of RIPK2 resulted in decreased HCMV replication. Given our findings of changes in IFN-β levels as a result of NOD2 KD or overexpression, it is possible that RIPK2-induction by NOD2 activates both the classical and alternative pathways in HCMV-infected cells. HCMV infection was reported to activate IRF3, a process that required STING, an endoplasmic reticulum-resident protein involved in DNA sensing [14]. In the case of Helicobacter pylori, RIPK2 induction by NOD1, activated IKKε and IRF7, followed by the synthesis of type I IFN and signaling of the later through IFN-stimulated gene factor 3 (ISGF3) [24]. Mycobacterium tuberculosis activated NOD2- RIPK2, which stimulated the activity of IRF5 and induced transcription of IFNα/β [43]. NOD2 activation in HCMV-infected cells appears to induce NF-_K_B and IFN signaling pathways at least in part through RIPK2. Taken together, NOD2 may act as a central PRR, but the downstream signaling pathways are pathogen-determined and governed by specific virus and cellular components. Additional studies will determine more specifically the downstream signaling pathways activated by NOD2 in HCMV-infected cells as well as potential interaction between NOD2 and TLR2

Episodes of HCMV colitis have been reported in patients with inflammatory bowel disease (IBD), both ulcerative colitis and Crohn's disease [46]–[48] and were thought to result from virus reactivation in patients receiving immunosuppressive therapy. The fact that NOD2 is a susceptibility gene for Crohn's disease triggered our study for its potential role in HCMV recognition [20]. Since our results show that NOD2 mutation (3020insC) results in enhanced HCMV replication, it is possible that NOD2, a susceptibility gene for Crohns's disease, may influence susceptibility to HCMV infection. Although not tested here, based on our data, we suggest that HCMV colitis in patients with Crohn's disease could represent a specific outcome of virus-host interaction in a subset of patients that carry mutations in the NOD2 gene. Additional studies using epithelial cells and clinical material will be required to prove our hypothesis. Although NLRs have been traditionally thought to sense bacterial pathogens, our data suggest a wider role for NOD2 in sensing viruses including persistent DNA viruses such as HCMV. In vivo studies are needed to confirm these findings and to elucidate the role of HCMV in the intestinal microbiome, for which NOD2 is a key regulator linking it to mucosal immunity [49].

## Supporting Information

Figure S1 **HSV1 and HSV2 do not induce NOD2 mRNA expression. A,** **B.** HFFs were infected with HSV1 KOS/Dlux/oriS (MOI = 1) or a clinical isolate of HSV2 (MOI = 0.1) and levels of NOD1 were determined by qRT-PCR at indicated time points. NOD2 levels were undetected in HSV1- and HSV2-infected cells. Quantitative data represent mean values (±SD) of triplicate determinations from two independent experiments.
(TIF)

Figure S2 **Knockdown of NOD2 results in enhanced HCMV replication in U373 cells. A**. U373 cells stably expressing control lentiviral vector (U373-GIPZ) or a lentiviral vector expressing short-hairpin RNA (shRNA) against NOD2 (U373-shNOD2) were infected with HCMV at MOI = 1, and levels of NOD2 mRNA were measured using qRT-PCR at 72hpi. **B.** Luciferase activity in cell lysates (measured at 96 hpi), virus DNA replication in supernatants (quantified at 96 hpi) and viral DNA replication (quantified at 48 hpi) were determined in cells from 5**A**. **C, D.** IFN-β and IL8 transcripts were measured in non-infected and HCMV- infected U373-GIPZ and U373-shNOD2 cells using qRT-PCR at 72 hpi. The data shown are the average of three experiments ± SD (*p<0.05, **p<0.01, ***p<0.001, one-way ANOVA test).
(TIF)
