# Supplementary figures and images for: Activation of Nucleotide Oligomerization Domain 2 (NOD2) by Human Cytomegalovirus Initiates Innate Immune Responses and Restricts Virus Replication
Source: PLoS One. 2014 Mar 26;9(3):e92704. doi: 10.1371/journal.pone.0092704 (PMC3966837; doi:10.1371/journal.pone.0092704)

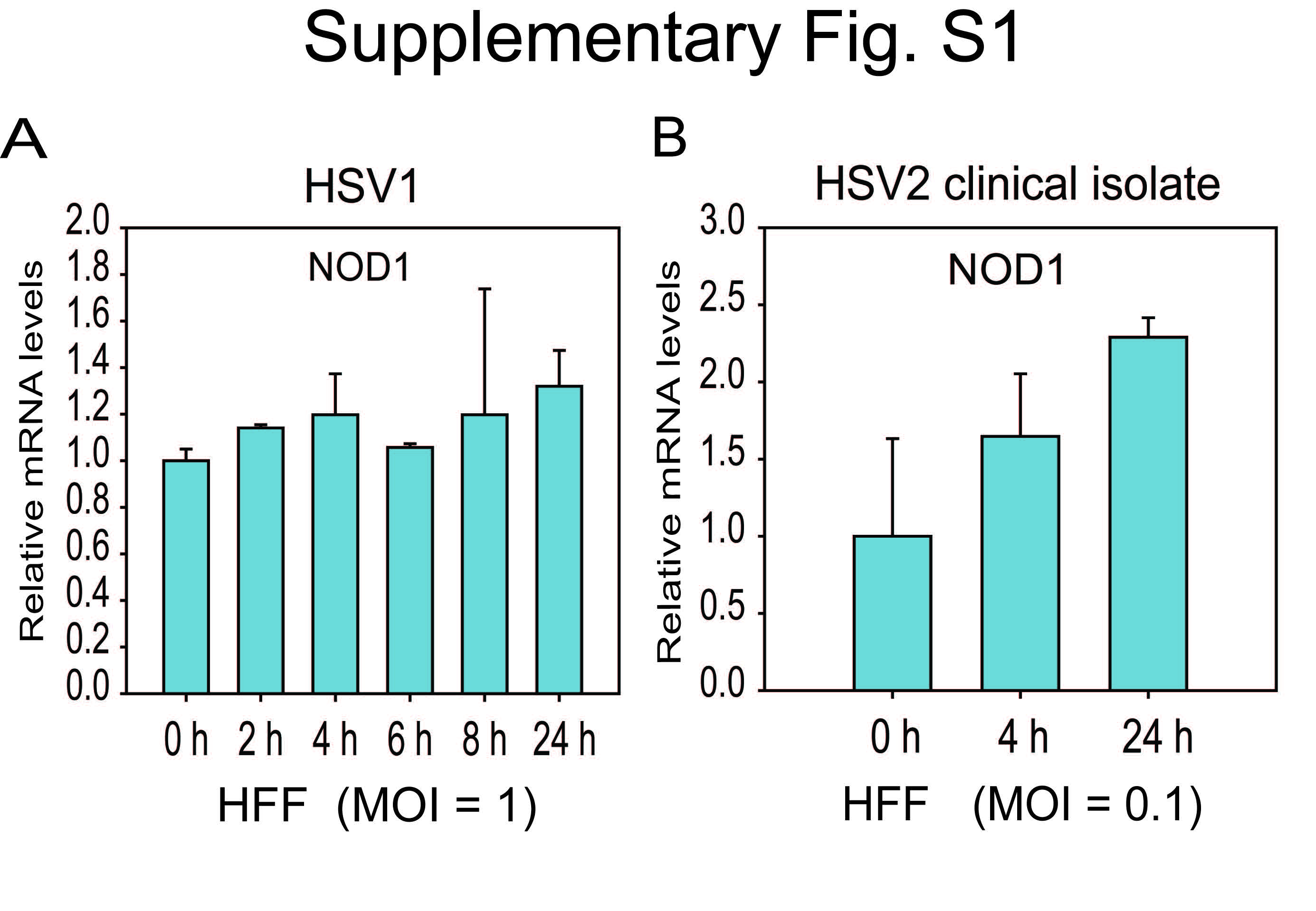

Supplement: Figure S1 — HSV1 and HSV2 do not induce NOD2 mRNA expression. A, B. HFFs were infected with HSV1 KOS/Dlux/oriS (MOI = 1) or a clinical isolate of HSV2 (MOI = 0.1) and levels of NOD1 were determined by qRT-PCR at indicated time points. NOD2 levels were undetected in HSV1- and HSV2-infected cells. Quantitative data represent mean values (±SD) of triplicate determinations from two independent experiments. (TIF) [file pone.0092704.s001.tif]

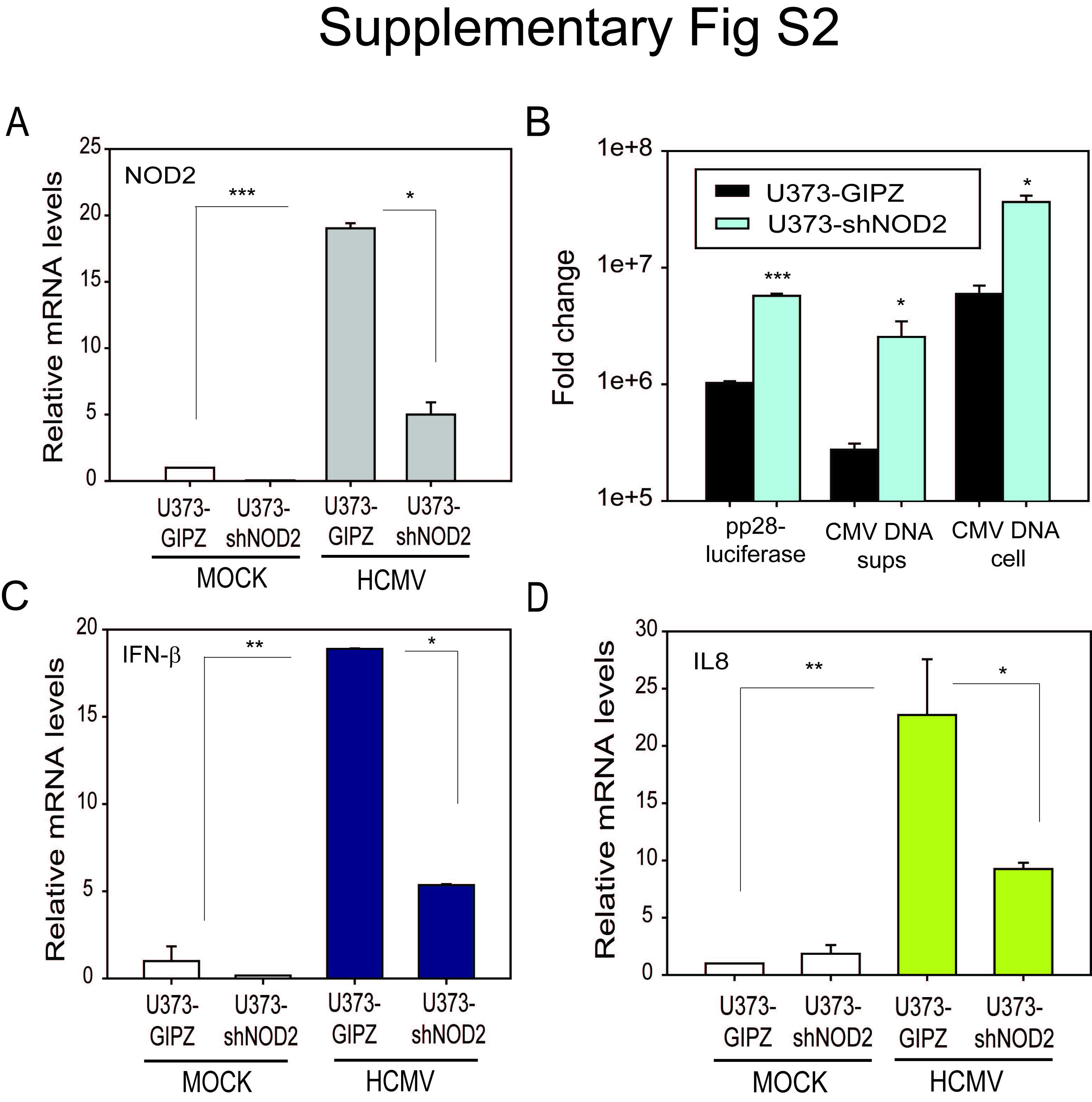

Supplement: Figure S2 — Knockdown of NOD2 results in enhanced HCMV replication in U373 cells. A. U373 cells stably expressing control lentiviral vector (U373-GIPZ) or a lentiviral vector expressing short-hairpin RNA (shRNA) against NOD2 (U373-shNOD2) were infected with HCMV at MOI = 1, and levels of NOD2 mRNA were measured using qRT-PCR at 72hpi. B. Luciferase activity in cell lysates (measured at 96 hpi), virus DNA replication in supernatants (quantified at 96 hpi) and viral DNA replication (quantified at 48 hpi) were determined in cells from 5A. C, D. IFN-β and IL8 transcripts were measured in non-infected and HCMV- infected U373-GIPZ and U373-shNOD2 cells using qRT-PCR at 72 hpi. The data shown are the average of three experiments ± SD (*p<0.05, **p<0.01, ***p<0.001, one-way ANOVA test). (TIF) [file pone.0092704.s002.tif]
